# Study on the Mechanism of Mesaconitine-Induced Hepatotoxicity in Rats Based on Metabonomics and Toxicology Network

**DOI:** 10.3390/toxins14070486

**Published:** 2022-07-14

**Authors:** Qian Chen, Kai Zhang, Mingjie Jiao, Jiakang Jiao, Dongling Chen, Yihui Yin, Jia Zhang, Fei Li

**Affiliations:** 1 School of Chinese Materia Medica, Beijing University of Chinese Medicine, Liangxiang Town, Fangshan District, Beijing 102488, China; chenqian19980101@foxmail.com (Q.C.); jmj9898@163.com (M.J.); jiaojiak123@163.com (J.J.); cdl181136@163.com (D.C.); 20190935005@bucm.edu.cn (Y.Y.); zhangjbucm@163.com (J.Z.); 2 School of Life Sciences, Beijing University of Chinese Medicine, Liangxiang Town, Fangshan District, Beijing 102488, China; zk006086@163.com

**Keywords:** mesaconitine, hepatotoxicity, metabonomics, network toxicology, oxidative stress, inflammatory response, apoptosis

## Abstract

Mesaconitine (MA), one of the main diterpenoid alkaloids in Aconitum, has a variety of pharmacological effects, such as analgesia, anti-inflammation and relaxation of rat aorta. However, MA is a highly toxic ingredient. At present, studies on its toxicity are mainly focused on the heart and central nervous system, and there are few reports on the hepatotoxic mechanism of MA. Therefore, we evaluated the effects of MA administration on liver. SD rats were randomly divided into a normal saline (NS) group, a low-dose MA group (0.8 mg/kg/day) and a high-dose MA group (1.2 mg/kg/day). After 6 days of administration, the toxicity of MA on the liver was observed. Metabolomic and network toxicology methods were combined to explore the effect of MA on the liver of SD rats and the mechanism of hepatotoxicity in this study. Through metabonomics study, the differential metabolites of MA, such as L-phenylalanine, retinyl ester, L-proline and 5-hydroxyindole acetaldehyde, were obtained, which involved amino acid metabolism, vitamin metabolism, glucose metabolism and lipid metabolism. Based on network toxicological analysis, MA can affect HIF-1 signal pathway, MAPK signal pathway, PI3K-Akt signal pathway and FoxO signal pathway by regulating ALB, AKT1, CASP3, IL2 and other targets. Western blot results showed that protein expression of HMOX1, IL2 and caspase-3 in liver significantly increased after MA administration (*p* < 0.05). Combined with the results of metabonomics and network toxicology, it is suggested that MA may induce hepatotoxicity by activating oxidative stress, initiating inflammatory reaction and inducing apoptosis.

## 1. Introduction

Mesaconitine (MA) is a C_19_-diterpenoid alkaloid derived from Aconitum, which has extensive biological activities and potential toxicity [1]. MA was considered to be one of the most active ingredients of Aconitum alkaloids, with analgesic, anti-inflammatory, positive inotropic, antiepileptic, antidepressant and vasodilating effects [2,3,4,5,6,7,8,9,10,11]. However, MA is a highly toxic ingredient, and this led to the controversy around using Aconitum herbs (such as *Radix Aconiti Lateralis*), which contain MA, in clinical setting. Therefore, it is necessary to systematically study the toxic mechanism of MA to provide reference for the safe clinical use of Aconitum.

At present, the toxicity studies on *Radix Aconiti Lateralis* (Fuzi) are mainly focused on its effect on the heart and central nervous system [12,13,14,15], and there are few reports on the hepatotoxicity mechanism of Fuzi. However, in recent years, the hepatotoxicity of traditional Chinese medicine has been widely discussed. Studies have shown that liver injury involves oxidative stress, inflammatory response, endoplasmic reticulum stress, apoptosis, cholestasis, immune function and other pathways. Flavonoids of vine tea may promote oxidative stress and pro-oxidative generation of free radicals and attack the mitochondria at the cellular level, causing cytotoxicity, triggering apoptotic mechanisms and causing damage to the liver [16]. Inflammasomes are a group of cellular protein complexes, which are closely related to liver diseases. They can recognize exogenous microorganisms, endogenous danger signals and different stressors, and then activate caspase-1 to produce IL-1β and IL-18 to initiate inflammation [17]. Studies have shown that acute inflammatory response is related to MAPK activation, NF-κB signaling induction, NRLP3 inflammasome pathway activation and oxidative stress increase, and the absence of Fpr1 gene expression has a positive effect on inflammation [18]. Direct hepatotoxicity is usually attributable to apoptosis or necrosis. Apoptosis can occur via the extrinsic pathway triggered by the activation of death receptors, such as Fas, and the intrinsic mitochondrial pathway, activated by intracellular perturbations, such as endoplasmic reticulum stress and oxidative damage [19]. It is reported that dose-dependent hepatotoxicity of Fuzi has been clearly observed in preclinical studies [20]. In addition, liver damage has been reported in several toxicity studies of rodents after single or long-term oral administration of Fuzi extract [21,22]. Studies have shown that after oral administration of Fuzi, the level of toxic alkaloids in the liver is very high, which can partly explain the hepatotoxicity of Fuzi, but the mechanism is still unclear [23]. In previous research, our research group screened out 22 potential hepatotoxic ingredients of Fuzi which can induce liver injury in rats [24]. As one of the potential hepatotoxic components of Fuzi, MA needs to be further studied.

At present, the characterization methods of drug toxicity mainly include serum biochemical markers detection and histopathological observation. These methods have certain limitations, and they cannot detect complex and huge metabolites, so it is difficult to evaluate the toxicity of MA in a timely and comprehensive way. Metabonomics is a systematic study of the overall metabolic response to stimulation. The unique metabolic changes induced by drugs reveal tissue damage types and related toxic mechanisms. The concept of analyzing life phenomena from a holistic perspective is very consistent with the treatment ideal of traditional Chinese medicine and is applicable to the study of traditional Chinese medicine [25]. Ultra-high-performance liquid chromatography coupled with quadrupoles hybrid electrostatic field orbital trap high-resolution mass spectrometry (UPLC-Q-Exactive Orbitrap-MS) is cutting-edge detection technology developed in recent years. It combines the advantages of both ultra-high-performance liquid chromatography and high-resolution mass spectrometry, and has characteristics such as high resolution, good quality accuracy and strong qualitative and quantitative capabilities, which make metabonomics analysis more efficient, in depth and accurate. Based on the interaction of “toxicity-gene-target-drug”, network toxicology constructs a complex regulatory network to explore the potential toxic mechanism of the research object, which can explain the molecular basis of disease from a multidimensional perspective. Network toxicology provides the information of enzymes and key proteins in signal pathways, and metabolomics provides the final pathophysiological results. The combination of metabolomics and network toxicology technology can reveal the potential complex relationship between multiple metabolites and multiple targets, mutually verify and supplement from different levels and reveal the mechanism of toxicity [26].

Based on the above research status, this study combined non-targeted metabolomics and network toxicology technology to study the basic toxicity of MA. By analyzing the screened differential metabolites and targets related to hepatotoxicity, as well as the enriched pathway information, we found that the hepatotoxicity of MA may be related to oxidative stress, inflammation and apoptosis. This study not only provides data support for the toxicity study of MA, but also provides a reference for the toxicity study of Aconitum herbs.

## 2. Results

### 2.1. Manifestation of Hepatotoxicity Induced by MA

#### 2.1.1. Analysis of Body Weight and Organ Coefficient

**Figure 1:**
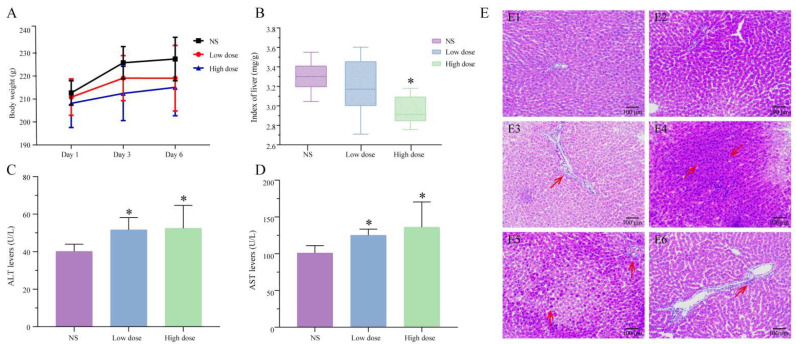
(**A**) The body weight of rats in the NS group and the MA group increased within 6 days. (**B**) Index of liver in NS group and MA group (compared with NS group: * *p* < 0.05). Results were analyzed by one-way ANOVA followed by a LSD test for multiple comparisons. (**C**) Serum ALT levels. The data were analyzed using a Kruskal–Wallis non-parametric test. (**D**) Serum AST levels. The data were analyzed using a Kruskal–Wallis non-parametric test. (**E**) Effects of MA on the morphology of liver in rats. NS group (**E1**,**E2**), low-dose group (**E3**,**E4**), high-dose group (**E5**,**E6**), red arrow points to hepatocyte necrosis or inflammatory cell infiltration.

As shown in Figure 1A, after 6 days of administration, compared with the NS group, the weight of rats in the low-dose group of MA tended to decrease, while the weight of the high-dose group decreased significantly (*p* < 0.05). The weight loss may have come from damage to organs caused by MA. The results of the organ coefficient (Figure 1B) showed that, compared with the NS group, the liver coefficient of the low-dose group of MA was decreased, and the liver coefficient of the high-dose group decreased significantly (*p* < 0.05). This suggested that the liver of the MA group may be damaged.

#### 2.1.2. Serum Biochemical Analysis

In this study, the levels of ALT and AST in rat serum were measured to evaluate the degree of liver injury. Results showed that compared with the NS group, the ALT and AST of low-dose and high-dose MA groups were significantly different (*p* < 0.05), and abnormal serum biochemical values indicated that the liver function of the administration group might be damaged (Figure 1).

#### 2.1.3. Analysis of Pathological Results

The histopathological results (Figure 1E) showed that the hepatocytes of the NS group were arranged radially around the central vein, with large and clear nuclei in the center. The structure of liver lobules was complete, and no clear liver injury was found. The sections of low-dose and high-dose groups of MA showed large necrotic areas, including lymphocytes, macrophages and neutrophils, which were large in volume and dark blue in basophilic color. There was inflammatory cell infiltration around the portal area, dense inflammatory cells gathered into pieces and granular degeneration was occasionally seen.

### 2.2. Metabonomics Study on Hepatotoxicity Induced by MA

#### 2.2.1. Methodology Test

In order to ensure the reliable quality of metabonomics data, the QC samples were tested for methodology. The same QC sample solution was continuously injected six times, and the data were exported as peak areas. RSD values of each ion peak area were calculated, and ions with RSD <30% accounted for 80.02%, indicating that the instrument was stable and had good precision; six QC sample solutions were prepared in parallel, and the samples were continuously injected for analysis. The data were exported as peak areas, and RSD values of each ion peak area were calculated. Ions with RSD <30% accounted for 85.67%, indicating that the method had good repeatability. The same QC sample solution was injected and analyzed at six time points in the whole injection sequence. The data were exported as peak areas, and RSD values of each ion characteristic were calculated. Ions with RSD <30% accounted for 80.08%, indicating that the stability of data collection was very good.

#### 2.2.2. Multivariate Statistical Analysis

**Figure 2:**
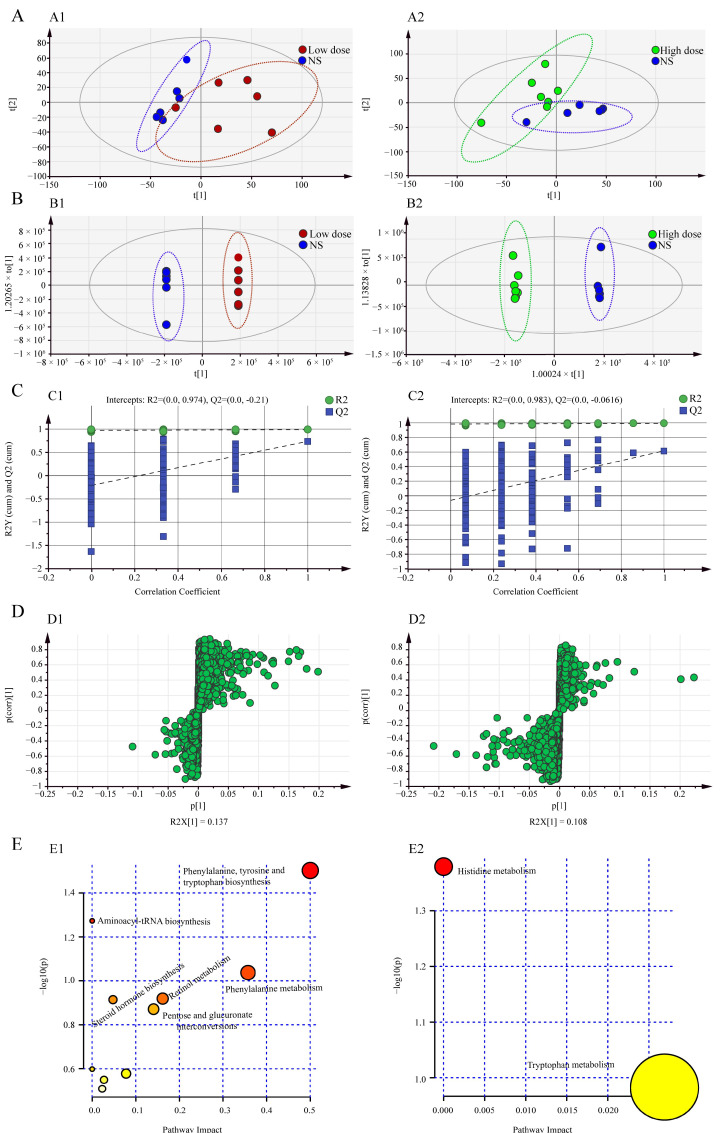
(**A**) PCA score plot for NS and MA groups. (A1: NS vs. low dose. A2: NS vs. high dose.). (**B**) OPLS-DA score plot for NS and MA groups. (B1: NS vs. low dose. B2: NS vs. high dose.). (**C**) Permutation test of OPLS-DA model. (C1: NS vs. low dose. C2: NS vs. high dose.). (**D**) S-plots obtained from OPLS-DA model. (D1: NS vs. low dose. D2: NS vs. high dose.). (**E**) Analysis of metabolic pathways. (E1: NS vs. low dose. E2: NS vs. high dose.).

The metabolic data were analyzed using PCA and OPLS-DA to explore the effect of MA on metabolism in rats. The data of each group were classified via PCA analysis, and the degree of aggregation and dispersion of samples could be observed. By analyzing the results of PCA (Figure 2A), it can be seen that MA can change endogenous metabolites in rats. The PCA scatterplots of NS group and MA administration group were separated. OPLS-DA is a supervised mode of analysis used to screen for differential metabolites. As shown in Figure 2B, the serum metabolites of NS group and the MA-administered group were completely separated, indicating that MA interfered with the metabolic profile of rats. Two hundred permutation tests were used to evaluate whether the OPLS-DA model was overfitted. The intercept of Q2 at the *y*-axis was less than zero, indicating that the model had not been overfitted. It was considered that the model was effective and reliable (Figure 2C).

#### 2.2.3. Screening of Differential Metabolites

The differential metabolites after administration of MA were screened by S-plots (Figure 2D) combined with the criteria of VIP ≥1 and *p* < 0.05, and the screened differential metabolites were identified using the HMDB database. A total of 17 differential metabolites were identified in the low-dose group and the NS group, mainly including L-phenylalanine, retinyl ester, aldosterone, L-proline, dehydroepiandrosterone, (4Z,7Z,10Z,13Z,16Z,19Z)-docosahexaenoic acid, β-D-glucuronoside, glycocholic acid, 5-hydroxyindoleacetaldehyde, etc. A total of 13 differential metabolites were identified in the high-dose group and the NS group, mainly including 5-hydroxyindoleacetaldehyde, methylimidazoleacetic acid, etc. (Table 1). By analyzing the differential metabolites between low-dose and high-dose groups of MA, six common differential metabolites were found, namely 5-Hydroxyindoleacetaldehyde, Indole-3-methyl acetate, Indole, MG (0:0/16:0/0:0), 3,5-Tetradecadiencarnitine and Gamma-linolenyl carnitine. Compared with the NS group, these six differential metabolites were upregulated in low-dose and high-dose MA groups, and Indole-3-methyl acetate, 3,5-Tetradecadiencarnitine and Gamma-linolenyl carnitine were upregulated more significantly in the high-dose group, while 5-Hydroxyindoleacetaldehyde, Indole and MG (0:0/16:0/0:0) were upregulated more significantly in the low-dose group. There were 11 unique differential metabolites in the low-dose group of MA, including L-Proline, L-Phenylalanine, Retinyl ester and β-D-Glucuronoside, etc., which were mainly related to amino acid metabolism, glucose metabolism and vitamin metabolism. There were seven unique differential metabolites in the high-dose group of MA, including 2-Hydroxymyristoylcarnitine, Dodecanoylcarnitine and Decanoylcarnitine, etc., which may be related to lipid metabolism.

#### 2.2.4. Metabolic Pathway Analysis

The screened differential metabolites were imported into Metabo Analyst 5.0 for metabolic pathway analysis, and the results are shown in Table 2. Trends of differential metabolites involved in metabolic pathways are shown in Table S1. As shown in Figure 2E, there were 10 metabolic pathways involved in differential metabolites between the low-dose group of MA and the NS group, mainly including phenylalanine, tyrosine and tryptophan biosynthesis, phenylalanine metabolism, retinol metabolism, arginine and proline metabolism, tryptophan metabolism and aminoacyl-tRNA biosynthesis, etc. The differential metabolites of the high-dose group and the NS group involved two metabolic pathways, namely tryptophan metabolism and histidine metabolism. By analyzing the metabolic pathways of low-dose and high-dose groups of MA, it was found that tryptophan metabolism was involved in both low-dose and high-dose groups. In addition, the low-dose group involved nine unique metabolic pathways, including phenylalanine metabolism, retinol metabolism, arginine and proline metabolism, etc. Histidine metabolism was a unique pathway in the high-dose group. The summarized metabolic pathways in the low-dose and high-dose rats are shown in Figure S1. It can be seen that MA can affect amino acid metabolism, vitamin metabolism, glucose metabolism and lipid metabolism.

### 2.3. Network Toxicological Study on Hepatotoxicity Induced by MA

#### 2.3.1. Acquisition of “MA-Targets”

A total of 299 targets were obtained from Pharm Mapper database, and 136 targets with Fit >3 were screened. The Swiss TargetPrediction database obtained 17 targets. After integrating and deduplicating the targets of two databases, 152 new aconitine targets were obtained.

#### 2.3.2. Acquisition of “Hepatotoxicity-Targets”

A total of 36 keywords related to hepatotoxicity were searched. By searching the keywords one by one, 10,250 genes with “marker/mechanism” were found in the CTD database, and 93,874 genes with “score > 40” were found in the GeneCards database. The details are shown in Table S2. A total of 552 targets were obtained after integration and deduplication of two online databases. At the same time, the chips were analyzed using R language: GSE103842, GSE113618, GSE116149, GSE119019, GSE129507 and GSE135079. In total, 84 differentially expressed genes were obtained. All the results were integrated and deduplicated, and finally, 634 hepatotoxic targets were obtained.

#### 2.3.3. Construction of Common Target Based on Pathoy Language

**Figure 3:**
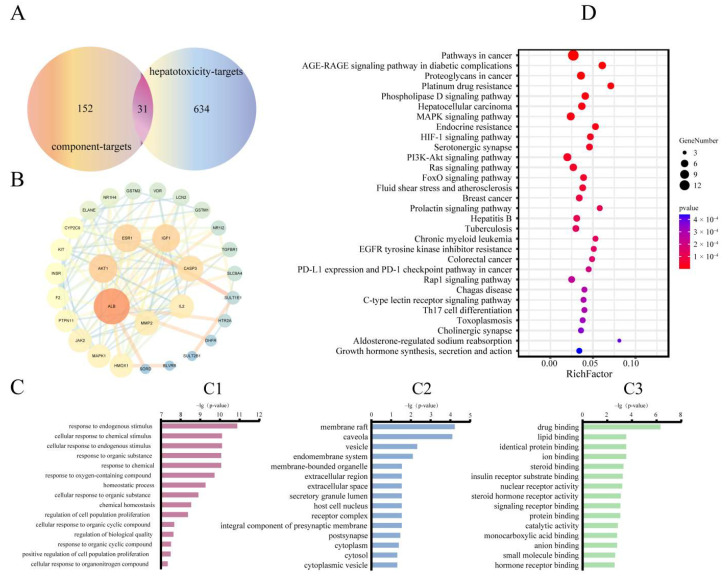
Analysis results of network toxicology. (**A**) Venn diagram of 31 potential “common targets” was intersected by “component targets” and “hepatotoxicity targets”. (**B**) Protein–protein interaction (PPI) network. (**C**) Top 15 items of Go analysis. (C1: biological process. C2: cellular component. C3: molecular function.) (**D**) Enrichment analysis of KEGG pathways.

The common targets of MA-induced hepatotoxicity in rats were introduced into pathoy, and 31 common targets were obtained. The results are shown in Figure 3A, and the information of these common targets is shown in Table S3.

#### 2.3.4. Construction and Analysis of PPI Network

The 31 common targets were imported into String for analysis, and the obtained results were imported into Cytoscape 3.7.2 for visualization. The results are shown in Figure 3B. The PPI network graph contains 30 nodes and 116 edges. The size and color of nodes are related to the “Degree” value. The targets with “Degree > 10” include ALB, AKT1, ESR1, IGF1, CASP3, IL2 and MMP2, and their nodes are larger and redder.

#### 2.3.5. Enrichment Analysis of GO and KEGG Pathways

The GO analysis results are shown in Figure 3C. The biological process enriched 491 items, mainly including “response to endogenous stimulus”, “cellular response to chemical stimulus”, “response to organic substance” and “response to oxygen-containing compound”. There were 15 cellular components, mainly including “membrane raft”, “caveola” and “vesicle”. There were 44 molecular functions, including “drug binding”, “lipid binding”, “identical protein binding”, “ion binding” and others. The KEGG pathway analysis results are shown in Figure 3D. A total of 122 pathways were enriched, and the top 30 pathways were selected for visual analysis according to the P value from small to large.

### 2.4. Effect of MA on Protein Expression of HMOX1, IL2 and Caspase-3 in Liver Tissue

**Figure 4:**
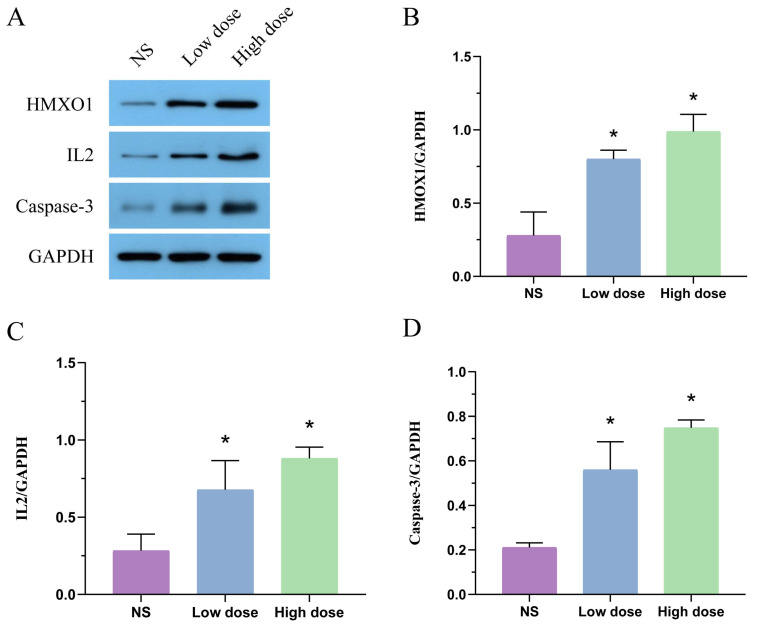
The protein expressions of HMOX1 (**A**,**B**), IL2 (**A**,**C**), caspase-3 (**A**,**D**) in liver tissue were detected by Western blot. Compared with the NS group, * *p* < 0.05.

As shown in Figure 4, compared with the NS group, the protein expression of HMOX1, IL2 and caspase-3 in the low-dose group and the high-dose group significantly increased after MA administration (*p* < 0.05).

## 3. Discussion

### 3.1. Manifestation of Hepatotoxicity Induced by MA

MA is a diester alkaloid derived from Aconitum, which possesses analgesic, anti-inflammatory and antiepileptic effects. At the same time, MA is also a highly toxic ingredient. The Median lethal dose (LD_50_) of MA in mice is 1.9 mg/kg, and the half-life is about 2.8−5.8 h [27]. In this study, after continuous administration of MA to rats for 6 days, the body weight and liver coefficient of the low-dose group were lower than those of the NS group, and the body weight and liver coefficient of the high-dose group were significantly different from the NS group (*p* < 0.05), suggesting that MA may cause liver damage in rats. ALT and AST are two membrane-bound enzymes related to liver dysfunction. The increase in ALT and AST levels in serum of rats treated with MA may be due to the increase in membrane permeability caused by oxidative stress, and ALT and AST are released from liver to serum [28]. In addition, according to the pathological sections of liver tissue, the treatment with MA resulted in typical hepatotoxic characteristics, such as hepatocyte necrosis and apoptosis, and infiltration of inflammatory cells around the portal area, indicating that MA has toxic effects on liver, which should be used with caution in clinical practice.

### 3.2. The Mechanism of Hepatotoxicity Induced by MA

According to the results of metabonomics and network toxicology, we found that MA may induce hepatotoxicity by activating oxidative stress, initiating inflammatory response and inducing apoptosis.

Oxidative stress is an unbalanced state in which reactive oxygen species (ROS) formation in vivo exceeds the antioxidant capacity of cells, resulting in the production of a large number of oxidative intermediates, inflammatory infiltration of neutrophils, increased secretion of protease and others. Our results showed that MA could affect the HIF-1 signaling pathway, increase protein expression of heme oxygenase 1 (HMOX1), downregulate the proline level and disorder tryptophan metabolism, resulting in oxidative stress damage. The HIF-1 signaling pathway enriched by network toxicology is one of the most important pathways involved in the regulation of oxygen homeostasis [28,29,30]. Hypoxia-inducible factor-1 (HIF-1) is a nuclear protein produced by cells under hypoxic conditions, in which HIF-1α subunit, as a key factor to respond to hypoxic stress, is regulated by hypoxic signals. HIF-1 can reduce mitochondrial stress by inhibiting mitochondrial division, improving mitochondrial oxygen metabolism, neutralizing ROS and regulating inflammatory response [31]. Studies have shown that stimulation of HIF-1 increases mitochondrial damage and accelerates liver injury [32]. Ubiquitination of HIF-1α is oxidation of its proline-by-proline hydroxylase, which is then recognized by ubiquitinase von Hippel–Lindau (VHL) for degradation. Blocked ubiquitination of HIF-1α can cause a series of oxygen homeostasis regulation responses in tissue cells. Proline is known as the elite of non-enzymatic antioxidants. Proline and hydroxyproline (metabolites of proline) have antioxidant properties that can scavenge reactive oxygen species, stabilize and upregulate antioxidant enzyme activity [33], improve cellular resistance to hydrogen peroxide and balance intracellular redox homeostasis, so proline is known as the elite of non-enzymatic antioxidants. Exogenous supplementation of L-proline can enhance the antioxidant capacity of rats [34]. The results of metabonomics showed that proline metabolism was disordered, and excessive production of ROS caused by downregulation of proline would lead to intracellular damage of biomolecules, including cell cycle blocking, autophagy and apoptosis [35]. HMOX1 is a downstream target gene of HIF-1α. HMOX1 is the most easily induced antioxidant enzyme in organisms discovered so far, and HMOX1 compensatory increases under oxidative stress [36]. The expression of HMOX1 in liver was significantly increased, suggesting that MA may cause oxidative stress injury and induce stress, increasing antioxidant protein HMOX1. 5-hydroxyindoleacetaldehyde is an intermediate metabolite of tryptophan metabolism, which can be biosynthesized from serotonin through the interaction with the enzyme kynurenine 3-monooxygenase. 5-hydroxyindoleacetaldehyde participates in many enzymatic reactions in the human body. Its upregulation caused by MA will inevitably affect the stability of tryptophan metabolism, cause oxidative stress and affect normal physiological functions [37,38].

Inflammation is a kind of defensive response of the body to stimulation, and current studies have clearly shown that hepatotoxic injury involves inflammatory response. Rhein exerts pro-inflammatory actions by increasing the interleukin-1 beta (IL-1β) and high-mobility group box-1(HMGB1) release, while downstream pro-inflammatory cytokines will, in turn, promote upstream intracellular signaling cascades and result in positive feedback pro-inflammatory signaling pathways. This amplifying mechanism of inflammatory signal pathways will eventually lead to further liver injury [39]. In this study, MA regulates inflammatory response through IL-2 and downstream PI3K-Akt signaling pathway and MAPK signaling pathway. Downregulation of phenylalanine metabolism and retinol metabolism disorder also proves that MA induces hepatotoxicity by initiating an inflammatory response. Interleukin-2 (IL-2), the target of network toxicology, is a cytokine that regulates innate and adaptive systems [40]. As a T-cell growth factor, IL-2 can promote proliferation of T cells and NK cells and promote B cells to produce antibodies and participate in immune response. Studies have shown that IL-2 can induce hepatic Kupffer cells, monocytes and macrophages to secrete TNF-α [41,42]. TNF-α causes direct cellular damage to liver by inducing production of chemokines, reactive oxygen species and adhesion molecules [43], and is a main inflammatory mediator of liver injury [44]. Western blot results show that IL-2, a cytokine related to inflammation, was upregulated in liver after MA administration, which mediates the inflammatory response. There are three main downstream signal pathways of IL-2, namely PI3K-AKT signal pathway, MAPK signal pathway and JAK-STAT signal pathway. PI3K can regulate NF-κB and its downstream proinflammatory cytokines [45,46,47]. Blocking PI3K/Akt will lead to an increase in NF-κB transcription and release of TNF-α, IL-1β and IL-6 [48]. Shen’s studies have shown that activation of PI3K/Akt signaling pathway increases expression of IL-4 and IL-10, decreases expression of IL-1β and TNF-α and reduces hepatic inflammatory response [49]. Studies have found that deletion of Akt1 and Akt2 in the liver can induce liver inflammation and hepatocyte death, and the use of PI3K/Akt inhibitors can significantly increase the degree of liver damage in mice [50]. In addition, the MAPK signaling pathway is also involved in inflammatory response [51]. Hydroxytyrosol (HT) administration is able to reduce colon inflammation, swelling, inflammatory cell infiltration and cytokine and chemokine overexpression [52], and HT increases the level of anti-inflammatory cytokines through the MAPK pathway [53]. Studies have shown that MAPK signaling cascade plays a key role in drug-induced hepatotoxicity by interacting with various signaling pathways [54,55,56,57,58]. Phenylalanine is an essential aromatic amino acid for the human body, which is very important for maintaining various functions and normal growth of the body. Phenylalanine is metabolized into tyrosine by the liver. However, when the liver is damaged, phenylalanine hydroxylase (PAH) will be destroyed, and the conversion of phenylalanine to tyrosine will be blocked, resulting in excessive phenylalanine concentration in the blood. Studies have shown that concentration of phenylalanine in plasma is positively correlated with the level of alanine aminotransferase (reflecting the degree of liver injury), and phenylalanine is a good biomarker for the severity of acute liver failure. Metabonomics results showed that phenylalanine was upregulated, indicating that liver function was impaired. Inflammation plays a key role in the increase in phenylalanine levels [59]. Inflammation-induced ROS production may consume a large part of tetrahydrobiopterin (BH4) [60]. BH4 is a cofactor of PAH, which directly affects activity of PAH, resulting in downregulation of the phenylalanine metabolism. The retinol metabolic pathway enriched by metabonomics is also closely related to liver. When liver is stimulated, retinoic acid can promote expression of RAE-1 (ligand of NKG2D receptor) in activated hepatic stellate cells and inhibit TGF-β/Smad3 signal, thereby improving the killing ability of NK cells and regulating inflammatory response.

Apoptosis is a spontaneous and orderly death, and its process is extremely complex. It is closely related to production of oxygen free radicals, disorder of cell energy metabolism, activation of cytokines, intracellular calcium overload and expression of cysteine protease (caspase) and B lymphocyte tumor-2 (Bcl-2) family genes. The disorder of apoptosis mechanism can induce a variety of diseases [61]. There are three main pathways of apoptosis, including mitochondrial apoptosis pathway, endoplasmic reticulum pathway and death receptor pathway. Mitochondrial pathway and endoplasmic reticulum stress belong to the endogenous pathway, and death receptor pathway belongs to the exogenous pathway. The endogenous pathway is mainly regulated by Bcl-2 family proteins, while the exogenous pathway is mainly regulated by caspase [62]. Caspase is a key mediator of programmed cell death or apoptosis, and it plays an important role in mediating apoptosis [63,64]. In this study, CASP3, the target of network toxicology, is the most important downstream effector molecule of orderly apoptosis cascade, and its activation is a sign of irreversible apoptosis [29]. Western blot results show that protein expression of caspase-3 in liver increased significantly after administration of MA, suggesting increased apoptosis. Activated caspase-3 can promote the cleavage of caspase-8 and amplify the pro-apoptotic signal pathway through mitochondria [65]. In addition, a large amount of evidence shows that the FoxO signaling pathway is also closely related to apoptosis, such as B cells, T cells, macrophages, neurons and glioma cells [66,67,68,69]. FoxO transcription factors belong to the Forkhead protein family, and their members include FoxO1, FoxO3, FoxO4 and FoxO6. One of the main expression sites of FoxO1 is hepatocytes, and its transcriptional regulation and signal transduction pathways are crucial for important physiological and pathological processes such as hepatocyte proliferation, apoptosis and cell cycle [70]. Studies have shown that reducing expression of p-FoxO1 can induce FoxO1 to enter the nucleus, thus activating the downstream apoptosis signal pathway [71]. The shuttle of FoxO between cytoplasm and nucleus is a key step in apoptosis [72]. In this study, CASP3 and FoxO signal pathway were obtained through network toxicology, and the target CASP3 was verified by Western blotting, which indicated that MA might induce hepatocyte apoptosis through them, thus leading to hepatotoxicity.

### 3.3. Limitations of the Study

This study combined metabolomics and network toxicology to comprehensively analyze the mechanism of MA-induced hepatotoxicity, but further research is still necessary. Firstly, there are some differences in the disturbance of endogenous metabolites between the high- and low-dose groups of MA, and the mechanism is not clear; secondly, the identification of endogenous differential metabolites in metabolomics completely depends on the online databases, which may lead to false positive results; finally, network toxicology is based on computer virtual computing and network database retrieval, and in vivo and in vitro experiments are necessary ways to further explore its mechanism. In this study, the targets of HMOX1, IL2 and CASP3 were verified by Western blotting. The systematic study of gene expression by transcriptomics and other techniques could further improve the mechanism of MA-induced hepatotoxicity. Based on the above points, future research will focus on a certain pathway for cell and animal experiments to further explore the hepatotoxicity mechanism of MA.

## 4. Conclusions

In conclusion, this study combined metabonomics and network toxicology to explore the hepatotoxicity mechanism of MA. Through metabonomics study, the differential metabolites of MA, such as L-phenylalanine, retinyl ester, L-proline and 5-Hydroxyindoleacetaldehyde, were obtained, which involved amino acid metabolism, vitamin metabolism, glucose metabolism and lipid metabolism. Based on the network toxicology analysis of online databases and multiple computer languages, 31 key targets of hepatotoxicity induced by MA, such as ALB, AKT1, CASP3 and IL2, were obtained. Western blot results show that protein expression of HMOX1, IL2 and caspase-3 in liver increased significantly (*p* < 0.05) after administration of MA. These targets were enriched by KEGG to obtain key cellular signal transduction pathways, such as HIF-1 signal pathway, MAPK signal pathway, PI3K-Akt signal pathway and FoxO signal pathway. The results of metabonomics and network toxicology further analyzed that MA might induce hepatotoxicity by activating oxidative stress, initiating inflammatory reaction and inducing apoptosis. The results provide data support for the toxicity study of MA and provide a reference for the toxicity study of Aconitum herb.

## 5. Materials and Methods

### 5.1. Materials and Animals

#### 5.1.1. Instruments

UPLC-Q-Exactive Orbitrap-MS (Thermo Fisher Scientific, Waltham, MA, USA); automatic biochemical analyzer (Beckman Coulter, Indianapolis, IN, USA); electronic balance (Sartorius, Gottingen, Germany); high-speed centrifuge (Thermo Fisher Scientific); vortex mixer (Dragon Laboratory Instruments Limited, Beijing, China); electrophoresis apparatus (Beijing Kaiyuan Xinrui Instrument Co., Ltd., Beijing, China).

#### 5.1.2. Reference Standards and Reagents

Mesaconitine (Batch number 110799-201608, China Institute for food and drug control); 4% paraformaldehyde tissue fixative (Beijing solabao Technology Co., Ltd., Beijing, China); sodium chloride injection (Shijiazhuang siyao Co., Ltd., Shijiazhuang, China); mass spectrometric formic acid (Thermo Fisher Scientific); mass spectrometric acetonitrile (Thermo Fisher Scientific); protease inhibitor cocktail (ROCHE); protein marker (Thermo). SDS-PAGE gel preparation kit, Ripa total protein lysate, BCA protein concentration determination kit, ECL chemiluminescence detection kit, TBS (powder), electrophoresis solution (powder), membrane transfer solution (powder), antibody eluent, Tween-20 and skimmed milk powder were all purchased from Wuhan baiqiandu Biotechnology Co., Ltd., Wuhan, China.

#### 5.1.3. Experimental Animals and Groups

Thirty-seven clean male SD rats weighing 180–200 g were purchased from SBF Biotechnology Co., Ltd. (Beijing, China) with the license number “SCXK (Jing) 2019-0010”. The rats were raised in the animal laboratory of Liangxiang campus of Beijing University of Chinese Medicine. They were raised under the following controlled environmental conditions: temperature 23 ± 2 °C, humidity 35 ± 5%, and 12 h day–night shift. Adaptive feeding was carried out for 7 days before the experiment, drinking water was provided for an unlimited time and feeding food was provided freely. Rats were randomly divided into three groups: high-dose group (*n* = 16, 1.2 mg/kg/day), low-dose group (*n* = 11, 0.8 mg/kg/day) and normal saline (NS) group (*n* = 10, 8.0 mL/kg/day). The administration scheme for rats is shown in Table 3.

All experimental procedures were conducted in accordance with China’s national legislation and local guidelines. The animal experiment was approved by the Animal Ethics Committee of Beijing University of Chinese Medicine (BUCM-4-2021071901-3023).

### 5.2. Manifestation of Hepatotoxicity Induced by MA

#### 5.2.1. Body Weight and Organ Coefficient

The rats were weighed and recorded before daily administration. After 6 days of administration, livers of all rats were weighed, and liver coefficients were calculated. The liver coefficient was calculated by dividing the weight of individual rat by the weight of the liver of that rat. The increase in the liver coefficient indicates that liver may have edema, hyperemia or hypertrophy; conversely, it indicates liver atrophy or other degenerative changes.

#### 5.2.2. Detection of Serum Biochemical Indexes

Rats were administrated continuously for 6 days. After fasting for 12 h, rats were anesthetized with 10% chloral hydrate at a dose of 3 mL/kg. Subsequently, 5 mL blood samples were collected from the abdominal aorta of rats. The whole blood was placed in a test tube and centrifuged at 3000 r/min and 4 °C for 15 min. The supernatant was centrifuged at 3500 r/min and 4 °C for 8 min. The serum obtained was used for serum biochemical detection and metabonomics research. Next, 150 μL was extracted from each serum sample, measured in an automatic biochemical analyzer, and the data of alanine aminotransferase (ALT) and aspartate aminotransferase (AST) were recorded.

#### 5.2.3. Histopathological Examination

The rat liver was removed and washed with normal saline to remove bloodstains. Filter paper was used to remove the surface water to dry the liver sample, which was then fixed in 4% polyformaldehyde solution. Paraffin sections of liver were prepared, and liver sections were stained with hematoxylin–eosin. The pathological manifestations were observed under a microscope for histological examination and evaluation.

### 5.3. Metabonomics Study on Hepatotoxicity Induced by MA

#### 5.3.1. Sample Preparation

##### Serum Sample Preparation

The serum samples stored in the −80 °C refrigerator were taken out and thawed at room temperature. Then, 450 μL of acetonitrile was added to 150 μL of serum, sonicated in an ice-water bath for 10 min, vortexed for 1 min, centrifuged at 13,000 rpm for 15 min at 4 °C and the supernatant was taken. The supernatant was blown dry with nitrogen, then 75 μL of 70% acetonitrile was added to redissolve, and followed by centrifugation at 13,000 rpm for 15 min at 4 °C. Finally, the supernatant was placed in a liquid phase vial with a lined tube.

##### QC Sample Preparation

All samples from the NS group and the administration group were drawn and mixed in equal amounts, and QC samples were prepared according to the method described in the Serum Sample Preparation part.

#### 5.3.2. Analysis Conditions

##### Chromatographic Conditions

C18 chromatographic column (Waters ACQUITY UPLC HSS T3 Column, polar endcapping, 2.1 mm × 100 mm, 1.8 µm); column temperature—30 °C; sample tray temperature—4 °C. Mobile phase A is 0.1% formic acid water and mobile phase B is acetonitrile. Elution conditions—0~3 min, 4~30% B; 3~7 min, 30~37% B; 7~10 min, 37~60% B; 10~21 min, 60~100%B; 21~21.5 min, 100% B; 21.5~22.5 min, 100~4% B; 22.5~26 min, 4% B. Flow rate—0.3 mL/min; sample volume—3 μL.

##### Mass Spectrometric Conditions

HESI ion source; positive ionization mode; ionization temperature—350 °C; scanning range—*m/z* 100–1500; primary scanning resolution—35,000; ionization source voltage—4 kV; capillary voltage—35 V; tube lens voltage—110 V; normalized collision energy—20%, 30%, 40%; sheath gas flow rate—40 arb; auxiliary gas flow rate—20 arb; both are nitrogen.

#### 5.3.3. Methodology Test

##### Instrument Precision

The same QC sample solution was continuously injected 6 times, and the data were exported as peak areas. RSD values of each ion peak area were calculated, and ions with RSD < 30% should account for more than 80%.

##### Method Reproducibility

Six QC sample solutions were prepared in parallel, and the samples were continuously injected for analysis. The data were exported as peak areas, and RSD values of each ion peak area were calculated. Ions with RSD < 30% should account for more than 80%.

##### Sample Stability

The same QC sample solution was injected and analyzed at 6 time points in the whole injection sequence. The data were exported as peak areas, and RSD values of each ion characteristic were calculated. Ions with RSD < 30% should account for more than 80%.

#### 5.3.4. Data Processing

MS-DIAL 4.70 software was used to perform non-linear correction in time domain and automatic integration and extraction of peak intensity for LC-MS data. Xcalibur 4.2 workstation was used to analyze and sort the data and control quality deviation range δ ≤ 5 × 10^−6^. According to the information of excimer ion peaks and ion fragments obtained by mass spectrometry, combined with literature search and relying on the HMDB database, metabolites were compared. Serum samples of each group were analyzed via principal component analysis (PCA) and partial least squares analysis (OPLS-DA) using SIMCA 14.1 software. Screening conditions: VIP ≥ 1 and *p* ≤ 0.05 as differential metabolites. MetaboAnalyst 5.0 was then used to enrich metabolic pathways.

### 5.4. Network Toxicological Study on Hepatotoxicity Induced by MA

#### 5.4.1. Acquisition of “MA-Targets”

Firstly, the two-dimensional structural formula of MA was obtained by PubChem (https://pubchem.ncbi.nlm.nih.gov/, accessed on 5 April 2022) and uploaded to PharmMapper (http://www.lilab-ecust.cn/pharmmapper/submitfile.html, accessed on 5 April 2022), which was used to obtain potential targets of MA. Then, the non-standard gene names obtained by Pharm Mapper were imported into Uniprot (http://www.uniprot.org/, accessed on 13 April 2022) to be converted into standard gene names, and the condition was defined as human in the popular organisms item. At the same time, we logged into Swiss TargetPrediction (http://www.swisstargetprediction.ch/, accessed on 13 April 2022), uploaded the two-dimensional structural formula of MA and obtained potential targets. Finally, the genes obtained from the two databases were integrated and de-duplicated, and MA-targets were obtained.

#### 5.4.2. Acquisition of “Hepatotoxicity-Targets”

We searched the keyword “liver injury” in PubMed (https://www.ncbi.nlm.nih.gov/, accessed on 1 March 2020) to obtain the most comprehensive keywords related to hepatotoxicity. Then, we logged on to CTD (http://ctdbase.org/, accessed on 1 March 2020) and Genecards (http://www.genecards.org/, accessed on 1 March 2020) to search the keywords related to hepatotoxicity one by one. The genes with “marker/mechanism” were screened using the CTD database, and the genes with “score > 40” were screened using the Genecards database.

In addition, the targets related to hepatotoxicity were mined through the GEO database. The gene expression profile chip data were imported into R language, data were cleaned and formatted and probe names were translated. Then, the differential expression genes were obtained using the linear regression model limma package. The setting conditions of “topTable” function: *p* < 0.05, logFC > 2.

Finally, all the obtained genes were integrated and de-duplicated to obtain “hepatotoxicity-targets”.

#### 5.4.3. Construction of Common Target Based on Pathoy Language

The data of MA-targets and hepatotoxicity-targets were imported into pathoy, and the “venn2” function under the “matplotlib_venn” module was used to draw the Wayne diagram of the two targets, and the common target—that is, the direct target of hepatotoxicity induced by MA in rats—was obtained by finding their intersection.

#### 5.4.4. Construction and Analysis of Protein–protein Interaction (PPI) Network

The potential hepatotoxic targets of MA were imported into the String database (https://stringdb.org/, accessed on 14 April 2022) to obtain the interaction data between genes, and the results were imported into Cytoscape 3.7.2 software for PPI network visualization. The topological analysis of PPI network was carried out by using the function “Network Analyzer”. The size and color of nodes in the interactive network were related to the “Dgree” of this node, that is, the larger the “Dgree” value of this node, the bigger the node and the redder its color. The width of an edge showed strength of interaction between the two nodes connected by this edge; that is, the stronger the interaction, the wider the edge.

#### 5.4.5. Gene Ontology (GO) Analysis and Kyoto Encyclopedia of Genes and Genomes (KEGG) Pathway Enrichment Analysis

GO and KEGG analysis of target genes in PPI network was carried out using the String database, and the files “enrichment.component”, “enrichment.Function”, “enrichment.Process” and “enrichment.KEGG” were obtained. GraphPad-Prism 6.0 and Omicshare (http://www.omicshare.com/tools/index.php/, accessed on 14 April 2022) were used to visualize these four files.

### 5.5. Western Blot Analysis

Proteins extracted from liver tissues of each group were used in Western blotting to validate the network toxicology results. The liver tissues were lysed using lysis buffer containing a protease inhibitor cocktail to extract total liver protein of rats in each group. The protein concentrations of samples were determined using the BCA method, and proteins were denatured via boiling for 5 min. Next, 40 μg of total protein in each group was loaded and separated using sodium dodecyl sulfate-polyacrylamide (baiqiandu) and transferred to methanol-activated polyvinylidene fluoride membranes (Millipore). Next, the membrane was blocked in TBST containing 5% skimmed milk for 1 h at room temperature, and incubated with primary antibody (HMOX1, 1:2000, Boster; IL2, 1:2000, Bioss; Caspase-3, 1:1000, Servicebio; GAPDH, 1:6000, Abcam) overnight, followed by incubation with the secondary antibodies (HRP-Goat anti rabbit, HRP-Goat anti mouse, 1:50,000, Searcare) at room temperature for another 30 min. ECL was used for protein imaging and development.

## Figures and Tables

**Table 1:** Identification results of potential differential metabolites in rat serum.

No.	Rt/min	Ion Mode	Obsed m/z	Calcd m/z	Error (ppm)	Formula	Identify	HMDB	Group
1	0.931	[M+H]+	130.08621	130.08625	−0.307	C6H11NO2	D-Pipecolic acid	HMDB0005960	High dose
2	1.918	[M+H]+	116.07054	116.07060	−0.517	C5H9NO2	L-Proline	HMDB0000162	Low dose
3	2.217	[M+H]+	132.10193	132.10190	0.227	C6H13NO2	D-Leucine	HMDB0013773	High dose
4	2.810	[M+H]+	115.11172	115.11174	−0.174	C7H14O	4-Heptanone	HMDB0004814	High dose
5	2.849	[M+H]+	166.08612	166.08625	−0.783	C9H11NO2	L-Phenylalanine	HMDB0000159	Low dose
6	3.373	[M+H]+	340.10211	340.10269	−1.705	C15H17NO8	5-Hydroxy-6-methoxyindole glucuronide	HMDB0010363	Low dose
7	3.886	[M+H]+	141.06564	141.06585	−1.489	C6H8N2O2	Methylimidazoleacetic acid	HMDB0002820	High dose
8	4.757	[M+H]+	514.28412	514.28329	1.614	C26H43NO7S	Sulfolithocholylglycine	HMDB0002639	Low dose
9	4.901	[M+H]+	176.07085	176.07060	1.420	C10H9NO2	5-Hydroxyindoleacetaldehyde	HMDB0004073	Low dose High dose
10	5.909	[M+H]+	190.08615	190.08625	−0.526	C11H11NO2	Indole-3-methyl acetate	HMDB0029738	Low dose High dose
11	6.336	[M+H]+	361.19955	361.20095	−3.876	C21H28O5	Aldosterone	HMDB0000037	Low dose
12	7.945	[M+H]+	466.31470	466.31631	−3.453	C26H43NO6	Glycocholic acid	HMDB0000138	Low dose
13	8.033	[M+H]+	289.21573	289.21620	−1.625	C19H28O2	Dehydroepiandrosterone	HMDB0000077	Low dose
14	8.034	[M+H]+	118.06486	118.06512	−2.202	C8H7N	Indole	HMDB0000738	Low dose High dose
15	8.536	[M+Na]+	351.23093	351.22945	4.214	C22H32O2	Docosahexaenoic acid	HMDB0002183	Low dose
16	8.823	[M+K]+	369.24194	369.24016	4.821	C19H38O4	MG (0:0/16:0/0:0)	HMDB0011533	Low dose High dose
17	9.351	[M+H]+	314.23276	314.23258	0.573	C17H31NO4	9-Decenoylcarnitine	HMDB0013205	Low dose
18	10.480	[M+H]+	316.24741	316.24823	−2.593	C17H33NO4	Decanoylcarnitine	HMDB0000651	High dose
19	12.718	[M+H]+	368.27847	368.27953	−2.878	C21H37NO4	3,5-Tetradecadiencarnitine	HMDB0013331	Low dose High dose
20	12.733	[M+H]+	388.30527	388.30574	−1.210	C21H41NO5	2-Hydroxymyristoylcarnitine	HMDB0013166	High dose
21	12.750	[M+H]+	295.22559	295.22677	−3.997	C18H30O3	9-OxoODE	HMDB0004669	Low dose
22	13.111	[M+H]+	344.28049	344.27953	2.788	C19H37NO4	Dodecanoylcarnitine	HMDB0002250	High dose
23	14.109	[M+H]+	303.23233	303.23185	1.583	C20H30O2	Retinyl ester	HMDB0003598	Low dose
24	18.521	[M+H]+	422.32602	422.32648	−1.089	C25H43NO4	Gamma-linolenyl carnitine	HMDB0006318	Low dose High dose

**Table 2:** Analysis results of metabolic pathways related to differential metabolites.

No.	Metabolic Pathway	Metabolite	p	−Log(p)	Holm p	FDR	Impact	Group
1	Phenylalanine, tyrosine and tryptophan biosynthesis	L-Phenylalanine	0.031463	1.5022	1.0	1.0	0.5	Low dose
2	Histidine metabolism	Methylimidazoleacetic acid	0.041783	1.379	1.0	1.0	0.0	High dow
3	Aminoacyl-tRNA biosynthesis	L-Phenylalanine; L-Proline	0.05338	1.2726	1.0	1.0	0.0	Low dose
4	Phenylalanine metabolism	L-Phenylalanine	0.091683	1.0377	1.0	1.0	0.35714	Low dose
5	Retinol metabolism	Retinyl ester	0.12049	0.91906	1.0	1.0	0.16168	Low dose
6	Steroid hormone biosynthesis	Dehydroepiandrosterone; Aldosterone	0.12185	0.91416	1.0	1.0	0.0474	Low dose
7	Pentose and glucuronate interconversions	beta-D-Glucuronoside	0.13457	0.87104	1.0	1.0	0.14062	Low dose
8	Biosynthesis of unsaturated fatty acids	(4Z,7Z,10Z,13Z,16Z,19Z)-Docosahexaenoic acid	0.25235	0.59799	1.0	1.0	0.0	Low dose
9	Arginine and proline metabolism	L-Proline	0.26449	0.57759	1.0	1.0	0.0778	Low dose
10	Tryptophan metabolism	5-Hydroxyindoleacetaldehyde	0.28236 0.10443	0.5492 0.98117	1.0	1.0	0.02691	Low dose High dose
11	Primary bile acid biosynthesis	Glycocholic acid	0.31125	0.50689	1.0	1.0	0.02285	Low dose

**Table 3:** Administration scheme of MA.

Grouping	Number	Drug	Dose	Method of Administration	Exposure Period
NS	10	0.9% NaCl	8.0 mL/kg/day	p.o., Continuous administration	6 days
Low dose	11	MA	0.8 mg/kg/day	p.o., Continuous administration	6 days
High dose	16	MA	1.2 mg/kg/day	p.o., Continuous administration	6 days

## Data Availability

The data presented in this study are available in this article and Supplementary Materials.

## Supplementary Materials

The following supporting information can be downloaded at: https://www.mdpi.com/article/10.3390/toxins14070486/s1. Table S1: Trends of differential metabolites involved in metabolic pathways; Table S2: Results of hepatotoxicity targets on online database; Table S3: Potential direct targets of hepatotoxicity induced by MA. Figure S1: The summarized metabolic pathways in the low 27 dose and high dose rats.
