# Supplementary material for: Study on the Mechanism of Mesaconitine-Induced Hepatotoxicity in Rats Based on Metabonomics and Toxicology Network
Source: Toxins (Basel). 2022 Jul 14;14(7):486. doi: 10.3390/toxins14070486 (PMC9322933; doi:10.3390/toxins14070486)
Supplement: Supplementary file 1 [file toxins-14-00486-s001.zip › toxins-1786076-supplementary.pdf]

toxins

MDPI

Supplementary Materials: Study on the Mechanism of Mesaconitine-Induced Hepatotoxicity in Rats Based on Metabonomics and Toxicology Network

Qian Chen, Kai Zhang, Mingjie Jiao, Jiakang Jiao, Dongling Chen, Yihui Yin, Jia Zhang and Fei Li

Contents:

Table S1 Trends of differential metabolites involved in metabolic pathways

Table S2 Results of hepatotoxicity targets on online database

Table S3 Potential direct targets of hepatotoxicity induced by MA

Figure S1 The summarized metabolic pathways in the low dose and high dose rats

Table S1 Trends of differential metabolites involved in metabolic pathways

Metabolic pathway	Metabolite	Trend （MA vs NS）
Phenylalanine, tyrosine and tryptophan biosynthesis	L-Phenylalanine	↑
Histidine metabolism	Methylimidazoleacetic acid	↑
Aminoacyl-tRNA biosynthesis	L-Phenylalanine	↑
Aminoacyl-tRNA biosynthesis	L-Proline	↓
Phenylalanine metabolism	L-Phenylalanine	↑
Retinol metabolism	Retinyl ester	↑
Steroid hormone biosynthesis	Dehydroepiandrosterone	↑
Steroid hormone biosynthesis	Aldosterone	↑
Pentose and glucuronate interconversions	beta-D-Glucuronoside	↑
Biosynthesis of unsaturated fatty acids	(4Z,7Z,10Z,13Z,16Z,19Z)-Docosahexaenoic acid	↑
Arginine and proline metabolism	L-Proline	↓
Tryptophan metabolism	5-Hydroxyindoleacetaldehyde	↑
Primary bile acid biosynthesis	Glycocholic acid	↑

Table S2 Results of hepatotoxicity targets on online database

No	Keywords	Targets in CTD	Targets with "marker/mechanism"	Targets in GeneCards	Targets of "score> 40"
1	Chemically-Induced Liver Toxicity	83368	410	685	0
2	Chemically Induced Liver Toxicity	83368	410	4897	30
3	Chemically-Induced Liver Toxicities	83368	410	685	0
4	Liver Toxicities,Chemically-Induced	83368	410	685	0
5	Liver Toxicity, Chemically-Induced	83368	410	685	0
6	Toxicities,Chemically-Induced Liver	83368	410	685	0
7	Toxicity, Chemically-Induced Liver	83368	410	685	0
8	Drug-Induced Acute Liver Injury	83368	410	1724	17
9	Drug Induced Acute Liver Injury	83368	410	5020	100
10	Liver Injury, Drug-Induced, Acute	83368	410	1724	17
11	Acute Liver Injury, Drug-Induced	83368	410	1724	17
12	Acute Liver Injury, Drug Induced	83368	410	5020	100
13	Hepatitis, Toxic	83368	410	4722	6
14	Toxic Hepatitis	83368	410	4722	6
15	Hepatitides, Toxic	83368	410	19	0
16	Toxic Hepatitides	83368	410	19	0
17	Drug-Induced Liver Disease	83368	410	2272	22
18	Disease, Drug-Induced Liver	83368	410	2272	22
19	Diseases, Drug-Induced Liver	83368	410	2272	22
20	Drug Induced Liver Disease	83368	410	11663	170
21	Drug-Induced Liver Diseases	83368	410	2272	22
22	Liver Disease, Drug-Induced	83368	410	2272	22
23	Liver Diseases, Drug-Induced	83368	410	2272	22
24	Drug-Induced Liver Injury	/	/	1939	6
25	Drug Induced Liver Injury	/	/	5989	62
26	Drug-Induced Liver Injuries	/	/	1939	6
27	Injuries, Drug-Induced Liver	/	/	1939	6
28	Injury, Drug-Induced Liver	/	/	1939	6
29	Liver Injuries, Drug-Induced	/	/	1939	6
30	Liver Injury, Drug-Induced	/	/	1939	6
31	Liver Injury, Drug Induced	/	/	5989	62
32	Hepatitis, Drug-Induced	/	/	1918	1
33	Drug-Induced Hepatitides	83368	410	17	0
34	Drug-Induced Hepatitis	/	/	1918	1
35	Hepatitides, Drug-Induced	83368	410	17	0
36	Hepatitis, Drug Induced	/	/	7396	45

Table S3 Potential direct targets of hepatotoxicity induced by MA

No	Gene	Targets	Type	Dgree
1	ALB	Serum albumin	direct targets	25
2	AKT1	RAC-alpha serine/threonine-protein kinase	direct targets	16
3	IGF1	Insulin-like growth factor IA	direct targets	15
4	ESR1	Estrogen receptor	direct targets	15
5	CASP3	Caspase-3	direct targets	13
6	MMP2	72 kDa type IV collagenase	direct targets	11
7	IL2	Interleukin-2	direct targets	11
8	MAPK1	Mitogen-activated protein kinase 1	direct targets	10
9	HMOX1	Heme oxygenase 1	direct targets	10
10	JAK2	Tyrosine-protein kinase JAK2	direct targets	10
11	PTPN11	Tyrosine-protein phosphatase non-receptor type 11	direct targets	9
12	KIT	Mast/stem cell growth factor receptor	direct targets	8
13	INSR	Insulin receptor	direct targets	8
14	F2	Prothrombin	direct targets	8
15	CYP2C9	Cytochrome P450 2C9	direct targets	8
16	NR1H4	Bile acid receptor	direct targets	6
17	ELANE	Leukocyte elastase	direct targets	6
18	VDR	Vitamin D3 receptor	direct targets	5
19	GSTM2	Glutathione S-transferase Mu 2	direct targets	5
20	GSTM1	Glutathione S-transferase Mu 1	direct targets	5
21	LCN2	Neutrophil gelatinase-associated lipocalin	direct targets	5
22	NR1I2	Nuclear receptor subfamily 1 group I member 2	direct targets	4
23	SLC6A4	Transporter	direct targets	4
24	TGFBR1	TGF-beta receptor type-1	direct targets	4
25	HTR2A	Serotonin receptor 2A	direct targets	3
26	SULT1E1	Estrogen sulfotransferase	direct targets	3
27	DHFR	Dihydrofolate reductase	direct targets	2
28	SORD	Sorbitol dehydrogenase	direct targets	1
29	BLVRB	Flavin reductase	direct targets	1
30	SULT2B1	Sulfotransferase family cytosolic 2B member 1	direct targets	1
31	CHRM3	Muscarinic acetylcholine receptor M3	direct targets	0

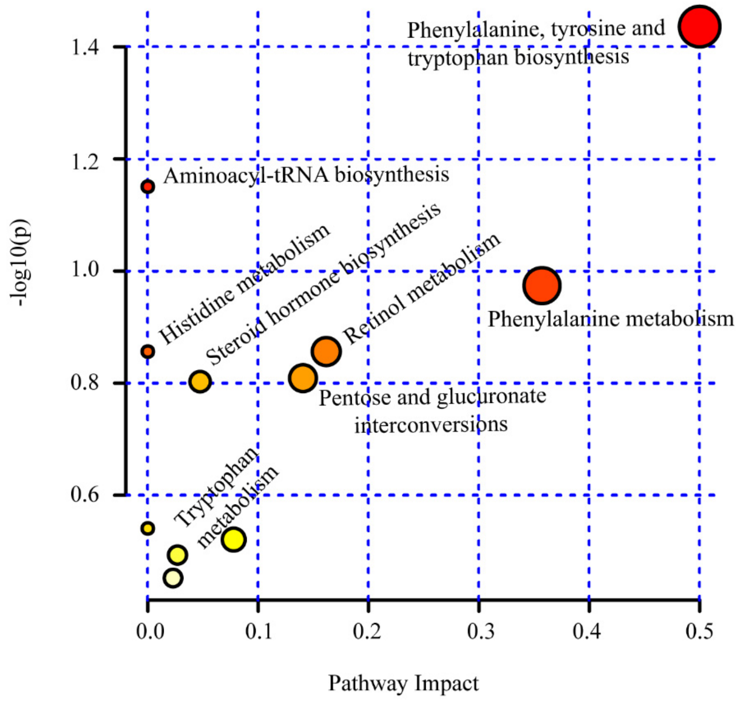

Figure S1. The summarized metabolic pathways in the low dose and high dose rats
